# Cognitive Advantages of Bilingual Children in Different Sociolinguistic Contexts

**DOI:** 10.3389/fpsyg.2017.00552

**Published:** 2017-04-21

**Authors:** Elma Blom, Tessel Boerma, Evelyn Bosma, Leonie Cornips, Emma Everaert

**Affiliations:** ^1^Special Education: Cognitive & Motor Disabilities, Department of Education & Pedagogy, Utrecht UniversityUtrecht, Netherlands; ^2^Amsterdam Center for Language and CommunicationAmsterdam, Netherlands; ^3^Fryske AkademyLeeuwarden, Netherlands; ^4^Faculty of Arts and Social Sciences, Maastricht UniversityMaastricht, Netherlands; ^5^Meertens InstituteAmsterdam, Netherlands

**Keywords:** bilingual advantage, dialect, bilingualism, regional language, minority language, working memory, attention

## Abstract

Many studies have shown that bilingual children outperform monolinguals on tasks testing executive functioning, but other studies have not revealed any effect of bilingualism. In this study we compared three groups of bilingual children in the Netherlands, aged 6–7 years, with a monolingual control group. We were specifically interested in testing whether the bilingual cognitive advantage is modulated by the sociolinguistic context of language use. All three bilingual groups were exposed to a minority language besides the nation’s dominant language (Dutch). Two bilingual groups were exposed to a regional language (Frisian, Limburgish), and a third bilingual group was exposed to a migrant language (Polish). All children participated in two working memory tasks (verbal, visuospatial) and two attention tasks (selective attention, interference suppression). Bilingual children outperformed monolinguals on selective attention. The cognitive effect of bilingualism was most clearly present in the Frisian-Dutch group and in a subgroup of migrant children who were relatively proficient in Polish. The effect was less robust in the Limburgish-Dutch sample. Investigation of the response patterns of the flanker test, testing interference suppression, suggested that bilingual children more often show an effect of response competition than the monolingual children, demonstrating that bilingual children attend to different aspects of the task than monolingual children. No bilingualism effects emerged for verbal and visuospatial working memory.

## Introduction

Over the past few years many studies have shown that bilingual children outperform monolinguals on tasks measuring executive functions (for reviews, see Adesope et al., 2010; Barac and Bialystok, 2011; Hilchey and Klein, 2011). The executive functions are part of a domain-general cognitive system that is essential for the flexibility and regulation of cognition and goal-directed behavior (Best and Miller, 2010) and comprises distinguishable yet interrelated components (Miyake et al., 2000). Commonly referred to components are switching (switching flexibly between tasks or mental sets), updating (constant monitoring and rapid addition/deletion of working-memory contents), and inhibition (control of attention and ability to override a strong prepotent response) (Miyake et al., 2000), although more recent insights suggest that inhibition may not be separable from the other components (Miyake and Friedman, 2012). Bilinguals are thought to develop executive function advantages because they manage multiple languages and continuously monitor the appropriate language for each communicative interaction (Costa et al., 2009). They need to attend to cues that inform the speaker on which language to use, select the right language, and choose appropriate lexicalisation, and while doing this they suppress the interference of other languages. As such, interactions in bilingual contexts may call upon general conflict-monitoring and goal-orienting abilities (Colzato et al., 2008; Costa et al., 2009; Hernández et al., 2013), leading to general executive function advantages (see Bialystok et al., 2012, for an overview).

However, other studies conclude that bilinguals’ better executive function outcomes are a mere artifact of experiments (Paap et al., 2015) or that bilingual cognitive advantages are attributable to factors such as differences in socioeconomic status (SES) between monolinguals and bilinguals (Morton and Harper, 2007). Other factors have also been proposed to account for the bilingual-monolingual differences in executive function tasks, such as general intelligence (Craik and Bialystok, 2005; Arffa, 2007; Brydges et al., 2012) or culture (Carlson and Choi, 2008; Carlson, 2009). To prevent confounding effects, within-group comparisons (Wu and Thierry, 2013; Crivello et al., 2016) and between-group comparisons in which the groups are matched on a range of background variables (Hilchey and Klein, 2011) are needed.

One study noteworthy for its careful design has been conducted by Duñabeitia et al. (2015). They compared the inhibitory skills of 252 monolingual Spanish and 252 Basque-Spanish bilingual children using a verbal and a numerical Stroop task in which children had to ignore distracting information. The monolingual children were recruited from provinces where Spanish is the only official language of communication. The bilingual children were recruited from the Basque country where both Basque and Spanish are official languages. Children in the two groups were matched on age, academic skills, attention-related skills, and intelligence. In this study no effects of bilingualism emerged on either of the two Stroop tasks. Furthermore, a large-scale study conducted with Welsh-English bilinguals showed little support for bilingual advantages on non-verbal executive function tasks (card sorting tasks, Simon tasks) and metalinguistic tasks (Gathercole et al., 2014). In both studies a relatively wide age range was included, but breaking down the results by grade or age group did not alter the conclusions.

Gathercole et al. (2014) suggest that it might not be coincidental that the Basque-Spanish and Welsh-English studies showed no effects of bilingualism, as the bilinguals in the two studies grew up in a situation of bilingual immersion in which both the minority language (Basque, Welsh) and the state’s official language (Spanish, English) are part of the everyday experience. Assuming a gradual approach to bilingualism, as for instance proposed by Dijkstra (2005) and Lam and Dijkstra (2010), they suggest that fluent bilinguals, such as the Basque-Spanish and Welsh-English bilinguals, have strong between-language links and a large degree of automaticity of the linguistic knowledge in both languages. Consequently, switching between languages may require little cognitive effort and control, and as a result, does not lead to the supposed training effect. Various other studies in which the bilinguals are also immersed in both the minority language and the state’s official language do show cognitive effects of bilingualism: balanced Frisian-Dutch children outperform Dutch-dominant children (Bosma et al., 2017), and Spanish-Catalan bilinguals (Costa et al., 2008, 2009; Hernández et al., 2013), Sardinian-Italian bilingual children (Lauchlan et al., 2013; Garraffa et al., 2015) and children who speak Cypriot Greek and Standard Modern Greek (Antoniou et al., 2016) outperform Spanish, Italian, and Greek monolinguals, respectively, on tasks testing executive functioning. It is possible that the participants in these studies are less bilingually fluent than the Basque-Spanish and Welsh-English bilinguals who showed no cognitive effects of bilingualism, because being immersed in the two languages does not necessarily imply fluent bilingualism. The hypothesis seems to be at odds, however, with observations showing that cognitive advantages are limited to bilinguals who are proficient in both languages (Carlson and Meltzoff, 2008; Bialystok and Barac, 2012; Poarch and van Hell, 2012; Videsott et al., 2012; Weber et al., 2016) or emerge as an effect of growing bilingual proficiency (Blom et al., 2014; Antoniou et al., 2016; Crivello et al., 2016).

Unlike the Basque-Spanish and Welsh-English bilinguals, the Frisian-Dutch, Catalan-Spanish, Sardinian-Italian bilinguals, and Cypriot Greek-Standard Modern Greek bidialectals are exposed to two closely related languages or dialects. This may suggest that language distance modulates the cognitive effect and that cognitive advantages are more likely for closely related varieties than for very distinct languages. However, there are also studies on closely related varieties that observe no cognitive effects suggesting that the relevant factor that might affect the emergence of the bilingual cognitive advantage in executive functioning is not language distance. For instance, no effects were observed in bilinguals who speak Italian and a Venetian regional variety (Scaltritti et al., 2015) or English and Dundonian, a regional variety spoken in the north-east of Scotland (Ross and Melinger, 2016). The conclusion that language distance does not modulate the cognitive effect of bilingualism is furthermore supported by research by Bialystok and colleagues who report effects of bilingualism on executive functioning in different language pairs (French-English, Chinese-English) and in heterogeneous bilingual samples (Bialystok, 1999; Bialystok and Martin, 2004; Bialystok et al., 2005). Comparing the Catalan-Spanish (Costa et al., 2008, 2009; Hernández et al., 2013) and Italian-Venetian contexts, Scaltritti et al. (2015) suggest that the frequency of language switching and mixing may explain the differential findings as the sociolinguistic environment of Catalan-Spanish bilinguals is conducive to language switching, whereas language use in the Italian-Venetian context is more compartmentalized. However, according to Green and Abutalebi (2013) separation may be more fundamental than frequency of switching, as functioning in settings in which the two languages are more separated is more likely to be associated with heightened cognitive control than functioning in dense code-switching contexts. The role of language separation appears to be supported by the findings of Antoniou et al. (2016) who observed cognitive effects in a context of diglossia in which the two language varieties (Cypriot Greek and Standard Modern Greek) are functionally separated and show hardly any overlap between domains of language use.

Despite the many studies that have investigated the cognitive effects of bilingualism, it is still not well-understood which conditions moderate the effect of bilingualism on executive functioning. With the present study, we aimed to shed more light on this issue by comparing three groups of 6- to 7-year-old bilingual children with a monolingual control group in the Netherlands. The four groups are matched on age, non-verbal intelligence, parental education, and gender. They differ in lingual status on two dimensions: besides the bilingual versus monolingual divide, the bilingual groups differ in exposure to a regional versus migrant language. In these two types of bilingualism, exposure to the minority language (i.e., regional, migrant) is not the same because exposure to the migrant language happens predominantly in the home environment, whereas the regional languages are also frequently spoken outside the children’s homes in the wider society. Children in the two regional language groups are exposed to either Frisian or Limburgish, apart from the national language (Dutch). Like Dutch, Frisian and Limburgish are West Germanic languages. Frisian and Limburgish share many linguistic properties with Dutch, but they are among the most linguistically distant from standard Dutch (Heeringa and Nerbonne, 2013). The sociolinguistic context is different for Frisian and Limburgish, which will be explained below.

Frisian is spoken in the province of Fryslân, in the north of the Netherlands, where it is an official language besides Dutch. As such, primary schools in the region are obliged to teach Frisian for at least 1 h per week, and in many schools Frisian is used as one of the languages of instruction. As Frisian has a long history of literacy and is present as language of instruction in education, Dutch and Frisian are considered and in general produced by its speakers as two separate varieties, even though code-mixing between Frisian and Dutch does happen regularly (Muysken, 2000). Frisian is recognized under part III of the European Charter for Regional and Minority Languages (ECRML), which went into force in 1998. This obliges the Dutch government to take concrete measures to promote Frisian in domains such as education, administration, and the media. In 2005, Frisian was recognized by the Dutch government as the only national minority language under the Framework Convention on the Protection of National Minorities. In 2014, the *Wet Gebruik Friese Taal* (‘Law on the use of the Frisian language’) went into force in the Netherlands, which states that Frisian and Dutch are the official languages of the province of Fryslân. Frisian has quite a strong position in the province, although it is more spoken in rural, than in urban areas (Breuker, 2001). In a recent survey of the province, slightly more than half of the population reported to speak Frisian as a mother tongue (55.3%) and slightly less than half of the population reported to speak Frisian with their partner (45.6%) and children (47.5%). Frisian is more used orally than written: most inhabitants of the province of Fryslân reported speaking it well (66.6%), but only few reported writing it well (14.5%) (Provinsje Fryslân, 2015).

Limburgish is spoken in the province of Limburg, in the south of the Netherlands. The dialects of Dutch Limburg were extended minor recognition under the label *Limburgish* in 1997 by the Netherlands, a signatory of the 1992 ECRML. Minor recognition under ECRML compels the Dutch state to formally recognize the status of Limburgish as a separate variety without, however, being obliged to take relevant measures such as financial support. Moreover, Limburgish is not taught in schools and, hence, it does not have the same status as Frisian. Since 1997, public funds have been made available by the Province of Limburg for promoting the use of Limburgish, although most people in Limburg, if not all, use the label dialect instead of Limburgish. Its use in local media is restricted (Cornips et al., 2016), as is its use in writing, public speech, educational contexts, and administration although, in contrast to Frisian, it is spoken as much in rural as in urban area’s (with the exception of the former coal mining area in the south-east). Although use of Limburgish is often limited to homely matters of family and community life (Leerssen, 2006) such as in the street, and in shops, it is also commonly spoken in formal domains, for instance by the highest-ranking provincial dignitaries and policy-officers in the provincial government building (Cornips et al., 2016). Therefore, it has a high social prestige in some societal and cultural domains. According to research conducted by the newspaper *De Limburger/Limburgs Dagblad* of a representative sample of 1,078 respondents in spring 2016, 66% indicated to be exposed to Limburgish from birth onward and 9% claimed to be raised partly in Limburgish. Moreover, 59% of the respondents claimed to be highly proficient in speaking the dialect of their birthplace and, in addition, 46 and 69% reported to be highly proficient in speaking and understanding the dialect of the village where they currently live in, respectively. Limburgish is spoken most with one’s own partner (64%) or children (62%) at home, with one’s parents (66%), and with friends (71%). In contrast, Dutch is the dominant language at the workplace or at school (53%), in civil services (65%), and in the hospital (75%). Although Limburgish and Dutch are perceived as two separate varieties, people frequently code-mix or speak a leveled variety between Dutch and Limburgish dialect in daily contexts (Giesbers, 1986).

The third bilingual group consists of Polish-Dutch immigrant children. Since 2004, when Poland entered the European Union, there has been an increase of Polish labor immigrants in the Netherlands. Recent demographic statistics indicate that there are 137,794 Polish immigrants in the Netherlands. The majority is first generation immigrant (78%) (Centraal Bureau voor de Statistiek [CBS], 2015). In general, Polish immigrants in the Netherlands have a higher educational level than the four largest immigrant groups (that is, migrants from Morocco, Netherlands Antilles and Aruba, Suriname, Turkey), and particularly Polish women are relatively well-educated (Dagevos, 2011). In our sample, the educational level of the Polish group was even higher than expected, which allowed us to match the four groups on SES. Because the influx of immigrants from Poland is relatively recent, limited information is available on language abilities and use in this group. The study by Dagevos (2011) reports that most Polish immigrants have a low level of Dutch and a good command of Polish. Both the low level of Dutch and high level of Polish are most probably related to recency of migration and are expected to change as a function of length of stay in the Netherlands. A minority of the Polish immigrants reports to always speak Dutch with their partner (20%) or children (10%). The reason why more Dutch is used with partners than children is twofold: relatively many Polish immigrants are in mixed marriages and many do not yet have children who are born in the Netherlands. It may be expected that use of Dutch in the Polish migrant families will increase when more children are born and educated in the Netherlands.

Executive function tasks in this study tested attention and working memory. Working memory refers to the ability to retain information in an accessible state (Engle, 2002). Various studies have shown that performance on working memory tasks is strongly related to attention, specifically to attentional control (Engle and Kane, 2004) and focus (Cowan et al., 2005). Attention and working memory are, however, not isomorphic and are best represented by correlated but distinct factors (Unsworth and Spillers, 2010). Previous research has shown bilingual advantages in attention (Martin-Rhee and Bialystok, 2008; Engel de Abreu et al., 2012), working memory (Morales et al., 2013; Blom et al., 2014) and in a combination of attention and working memory tasks (Antoniou et al., 2016), using similar tasks as those used in the present study. The attention tasks in the present study tested the ability to filter information and focus on task-relevant stimuli (selective attention) and the ability to suppress interference from a specific cue (interference suppression). For working memory, both verbal and visuospatial working memory tasks were used (Alloway et al., 2006).

We expected the bilingual children to outperform the monolingual children on working memory and attention, but because there are also studies reporting no effects, we reckoned with the possibility that no effects would emerge. There could also be reasons why some bilingual groups differ from the monolinguals whereas others do not. Exposure to Polish in a limited number of domains (because of in-home exposure only) may result in a relatively low degree of bilingual proficiency in the Polish group. Given previous observations that a certain level of bilingual proficiency is required for cognitive effects to develop (Carlson and Meltzoff, 2008; Bialystok and Barac, 2012; Poarch and van Hell, 2012; Videsott et al., 2012; Blom et al., 2014; Antoniou et al., 2016; Crivello et al., 2016; Weber et al., 2016), it is possible that the Polish group shows no bilingual benefit, in contrast to the Frisian and Limburgish groups. The Polish group is not as bilingually immersed as the Frisian and Limburgish groups, where both languages are used frequently inside and outside the home environment. Given this difference, children in the Frisian and Limburgish groups may be more fluent bilinguals and experience more overlap in the domains of language use than the Polish children. Fluent bilingualism (Gathercole et al., 2014) and limited language separation (Green and Abutalebi, 2013) may predict that the Frisian and Limburgish groups do not outperform the monolinguals on executive functioning, and contrast with the Polish group in this respect. Between the Frisian and Limburgish contexts, differences exist in use of the regional language in school settings. Teaching Frisian as a subject in school may contribute to language separation, because it raises awareness that Frisian is a separate language. This would predict that the Frisian children are more likely to show cognitive effects of their bilingualism than the Limburgish children. However, the use of Frisian as a language of instruction, besides Dutch, could have the opposite effect because functional overlap between the two languages in a specific domain, like the educational context, may lead to less separation instead of more.

## Materials and Methods

### Participants

In the study 176 children participated, who were assigned to four different groups (monolingual Dutch, Frisian-Dutch, Limburgish-Dutch, Polish-Dutch), with 44 children in each group. All children were either 6 or 7 years old (72–95 months) at time of testing. In addition to age, a number of selection criteria were used. Children with a non-verbal intelligence below 70 were excluded, as were children for whom full datasets were not available. Furthermore, within the bilingual groups, children were only included if at least one of their parents spoke the non-Dutch language with the child, to ensure that all these children could be considered bilingual. Details of the groups are given in **Table 1**.

**Table 1:** Mean age, NVIQ, SES, with standard deviations, and gender distribution in the four groups.

	N	Age in months	NVIQ	SES	Girls/boys
Monolingual	44	82 (7)	107 (15)	6.56 (1.94)	20/24
Frisian	44	82 (6)	107 (15)	6.73 (1.28)	20/24
Limburgish	44	84 (6)	108 (13)	6.72 (1.93)	20/24
Polish	44	82 (7)	108.5 (13)	7.28 (1.40)	22/22

*N, number; NVIQ, standardized non-verbal intelligence score; SES, socioeconomic status.*

The four groups did not differ in age [*F*(3,172) = 1.13, *p* = 0.34, $\eta_{p}^{2}$ = 0.02]. In addition to age, they were matched on non-verbal intelligence [*F*(3,172) = 0.20, *p* = 0.90, $\eta_{p}^{2}$ = 0.003], SES [*H*(3) = 3.71, *p* = 0.30, $\eta_{p}^{2}$ = 0.004], and gender [χ^2^(3) = 0.27, *p* = 0.97]. Non-verbal intelligence was measured with the short version of the *Wechsler Nonverbal-NL* (Wechsler and Naglieri, 2008), and SES was indexed by the average educational level of both parents of the child, based on the *Questionnaire for Parents of Bilingual Children* (PaBiQ; Tuller, 2015). Educational level represented the highest degree obtained on a nine-point scale ranging from 1 indicating no education to 9 indicating a university degree.

The Frisian-Dutch, Limburgish-Dutch, and monolingual Dutch participants were recruited via regular elementary schools in the north, south, and mid-west of the Netherlands, respectively. All these children were born in the Netherlands. The Polish-Dutch children were recruited via Polish Saturday schools in the western part of the country. In the Polish group, 70% of the children were born in the Netherlands (mean age of arrival = 8.62 months, *SD* = 20.38). All Polish children had lived in the Netherlands for 2 years or more at time of testing. Parental questionnaire data, collected with the PaBiQ (Tuller, 2015), indicated that the bilingual children had received a substantial amount of input in Dutch before the age of 4, measured relative to the total amount of language input that the child received before this age (both inside and outside home context). From the age of 4 onward, all children received regular and frequent exposure to Dutch in kindergarten. There was a difference between the bilingual groups with respect to Dutch input before age 4 [*F*(2,129) = 4.83, *p* = 0.009, $\eta_{p}^{2}$ = 0.07]. The Frisian group had received less Dutch exposure than the Polish (*p* = 0.01) children. There was no difference between the Frisian and the Limburgish group (*p* = 0.07) or between the Polish and the Limburgish group (*p* = 1.00). The PaBiQ also provided information on the current use of languages at home, measured relative to the total amount of language input that the child heard from its mother, father, siblings, and other adults that had frequent contact with the child. The groups did not differ on current use of Dutch [*F*(2,129) = 2.06, *p* = 0.13, $\eta_{p}^{2}$ = 0.03]. The language input and use patterns at home in the three bilingual groups can be found in **Table 2**.

**Table 2:** Mean input with standard deviations in different languages before age 4 and current use of languages at home.

	% Dutch input before age 4	% Non-Dutch input before age 4	% Other input before age 4	% Current use Dutch	% Current use non-Dutch	% Current use other
Frisian	30 (23)	70 (24)	1 (3)	31 (27)	68 (26)	0 (2)
Limburgish	40 (20)	59 (21)	1 (5.5)	42 (26.5)	56 (27)	1 (6)
Polish	43 (19)	54 (18)	3 (8)	37 (24)	61 (24)	1.5 (8)

*Non-Dutch refers to Frisian, Limburgish and Polish, respectively, whereas ‘other’ refers to additional other languages that the children are exposed to.*

As expected, the bilingual children were quite proficient in Dutch as confirmed by the outcomes of the Dutch version of the Peabody Picture Vocabulary Test (PPVT; Schlichting, 2005), which is a standardized measure for Dutch receptive vocabulary. The data in **Table 3** show that each group scored, on average, within the normal range of variation for monolingual Dutch children, that is, within 1 standard deviation from the mean of the normative sample (*M* = 100, *SD* = 15). In the Polish sample there were six children who scored 1 standard deviation below the mean and one child who scored over 2 standard deviations below the mean. Most of these children were not born in the Netherlands but arrived at a later age, explaining these relatively low scores. On average the Polish group also had a lower PPVT score than the other three groups as indicated by a univariate ANOVA [*F*(3,172) = 12.42, *p* < 0.001, $\eta_{p}^{2}$ = 0.18] and subsequent Bonferroni *post hoc* comparisons (monolingual: *p* < 0.001, Frisian: *p* < 0.001, Limburgish: *p* = 0.01). Further information about children’s skills in Dutch and the non-Dutch language was obtained through the PaBiQ. The Frisian and Polish parents indicated quite similar skills in both languages [Frisian: *t*(43) = -0.78, *p* = 0.44; Polish: *t*(43) = -1.55, *p* = 0.13], whereas the Limburgish parents reported that their children’s skills in Dutch were better than in Limburgish [*t*(40) = -6.06, *p* < 0.001].

**Table 3:** Mean Dutch receptive vocabulary (standardized) score and mean Dutch and non-Dutch language skills as indicated by parental report with standard deviations.

	Dutch receptive vocabulary score	Skills Dutch scale 0-1	Skills non-Dutch language scale 0-1
Monolingual	112 (12)	0.83 (0.17)	–
Frisian	109 (10)	0.76 (0.17)	0.73 (0.21)
Limburgish	106 (8)	0.91 (0.12)	0.59 (0.31)
Polish	98 (14)	0.73 (0.20)	0.65 (0.25)

*The receptive vocabulary score is a standardized score with a mean of 100.*

### Measures

#### Receptive Vocabulary

Receptive vocabulary in Dutch was measured with the *Peabody Picture Vocabulary Task* (PPVT-III-NL; Schlichting, 2005, based on the PPVT-III by Dunn and Dunn, 1997). The PPVT is a standardized receptive vocabulary test designed for the age range from 2 years and 3 months to 90 years. It contains 204 items divided over 17 sets. The sets are ordered according to difficulty and each set consists of 12 items. In this task, the child hears a stimulus word and has to choose the correct referent out of four pictures. The PPVT-III-NL was administered and scored according to the official guidelines. Receptive vocabulary in Polish was measured with the standardized *Obrazkowy Test Słownikowy – Rozumienie* (Haman and Fronczyk, 2012). This instrument is very comparable to the PPVT and offers monolingual norms for the age range from 2 to 6 years and 11 months. For Frisian and Limburgish, no standardized receptive vocabulary measures were available.

#### Parental Questionnaire

The *Questionnaire for Parents of Bilingual Children* (Tuller, 2015) was administered during a home visit or telephone interview with one of the child’s parents. For the bilingual children, the interview was conducted by bilingual assistants, who were proficient in both Dutch and Frisian/Limburgish/Polish and could therefore be carried out in the preferred language of the parent. For the monolinguals, the interview was in Dutch. The questionnaire administered to the parents of the monolingual children was a short version of the PaBiQ in which the items that were only relevant for bilingual children (e.g., amount of input in the different languages, skills in the non-Dutch language) were removed. As described under ‘Participants,’ the PaBiQ provided information on parental education, on the child’s language input before the age of 4, on the current language use at home, and on the child’s language skills as evaluated by the parent.

#### Working Memory

Verbal and visuospatial working memory were measured with a backward Digit Span task and a backward Dot Matrix task, respectively. These tasks were based on the *Alloway Working Memory Assessment* (AWMA; Alloway, 2012) and translated to Dutch by native to near-native speakers of Dutch and English. In the backward Digit Span task the children had to repeat sequences of auditorily presented digits in reverse order. In the backward Dot Matrix task the children were presented with sequences of blue dots that appeared in a 4 × 4 matrix on a computer screen. After the last dot disappeared, the children had to point out the position of the dots in reverse order. The tasks started with a block of six trials with sequences of one digit or dot, after which the difficulty level was gradually increased. The AWMA procedure was applied for scoring. One point was given for each correct trial, so there was a maximum of six points per block. Trials were scored as incorrect if children recalled one or more digits/dots incorrectly, if the sequence was incorrect, or if they omitted one or more digits/dots. Children automatically continued with the next block when they repeated the first four trials within one block correctly, in which case they received a score of 6, or when they repeated four of the first five trials within one block correctly, in which case they received a score of 5. The task stopped automatically when children responded incorrectly to three trials within the same block. There were six blocks in the Dot Matrix task and seven in the Digit Span task, so the scores could range from 0 to 36 for the Dot Matrix and from 0 to 42 for the Digit Span.

#### Attention

Selective attention was measured with the visual Sky Search task from the *Test of Everyday Attention for Children* (Manly et al., 1999). In the Sky Search task, children had to look for identical pairs of spaceships on an A3 sheet of paper. The test sheet contained 20 identical pairs and 108 non-identical pairs. The children had to encircle the identical pairs as fast as they could while ignoring the non-identical pairs. They indicated themselves when they were finished. The time per target (i.e., an identical pair of spaceships) was calculated, which was the time it took children to do the task divided by the total number of correctly encircled pairs of spaceships. Selective attention was measured because the children had to focus on the identical pairs in order to perform well on the task. After completion of this sheet the children were given a second A3 sheet, which was the motor-control version of the task. On this sheet only the 20 pairs of identical spaceships were displayed. Again, they had to encircle all the pairs of spaceships as fast as they could; the time per target was calculated for this sheet as well. Children’s selective attention score was then calculated by subtracting the time per target of the motor-control sheet (i.e., second sheet) from the time per target of the test sheet (i.e., first sheet). In this way drawing speed was controlled for.

Interference suppression was measured with a flanker test from Engel de Abreu et al. (2012), who adapted the child Attention Network Task from Rueda et al. (2004a,b). The task was administered on a laptop using the experimental software E-Prime 2.0 (Schneider et al., 2002). In the Flanker task a horizontal row of five equally spaced yellow fish was presented to the children. The children were asked to indicate the direction of the central fish by pressing the corresponding left or right response button as quickly as possible. On congruent trials (50%), the flanking fish were pointing in the same direction as the central target fish, and on incongruent trials (50%), the flanking distractors pointed in the opposite direction. Each trial started with a fixation cross in the middle of the screen (1000 ms), followed by the presentation of the five fish. Children had to respond by pressing a left or a right button within 5000 ms. A response after 5000 ms was considered incorrect. All children completed two blocks of 20 trials in which presentation of congruent and incongruent trials was randomized. Eight practice trials preceded the test phase. Accuracy and reaction times (RTs) were documented automatically through E-Prime. As accuracy scores were very high in all groups and in both the congruent and incongruent conditions (>95% correct), we decided to focus on RTs in the analyses. Following Engel de Abreu et al. (2012), mean RTs were calculated excluding incorrect responses, RTs below 200 ms and RTs above 3 standard deviations of children’s individual means (<5% of all trials). The flanker effect, reflecting the difference between the average RTs on incongruent and congruent trials (mean RT_INCONGRUENT_ – mean RT_CONGRUENT_), was used as our dependent variable. A small flanker effect was assumed to indicate good ability to suppress interference, whereas a large flanker effect was thought to indicate limited resistance to interference.

### Procedures

This research was screened by the Standing Ethical Assessment Committee of the Faculty of Social and Behavioral Sciences at Utrecht University. Criteria were met and further verification was not deemed necessary. Parents of participants gave informed consent in accordance with the Declaration of Helsinki. All participants were tested individually in a quiet room at their school or their home. The children completed a battery of tests, including several measures that tapped into language, working memory, and attention (not all relevant for the current study). The experimenters who administered the tests all had a native command of Dutch and, in the case of the bilinguals, also of Frisian, Limburgish, or Polish. The language of instruction for all relevant measures was Dutch, except for the Polish receptive vocabulary task, which was administered with Polish instructions.

## Results

### Comparisons between Groups

**Table 4** shows the results of the monolingual and bilingual groups on the working memory and attention tasks.

**Table 4:** Mean working memory and attention scores with standard deviations in the different groups.

	Backward Digit Span	Backward Dot Matrix	Sky Search	Flanker effect (in ms)
Monolingual	15.09 (2.66)	17.27 (4.83)	6.07 (2.77)	91.84 (194.77)
Bilingual	14.61 (2.90)	17.39 (5.21)	5.30 (2.20)	136.38 (207.52)
Frisian	14.80 (3.14)	17.89 (4.72)	4.85 (1.46)	142.81 (185.57)
Limburgish	15.25 (2.91)	17.20 (6.33)	5.17 (2.29)	86.03 (223.28)
Polish	13.80 (2.49)	17.07 (4.47)	5.88 (2.60)	180.30 (205.67)

In the case of the two working memory tasks (backward Digit Span, backward Dot Matrix) a higher score indicates better performance, but in the case of both attention tasks (Sky Search, Flanker) a higher score points to lower performance. The flanker effect showed substantial individual variation as indicated by the large standard deviations. Inspection of the individual scores revealed that these are caused by *negative* effects, indicating it took children longer to respond to the congruent than to the incongruent items. For reasons of interpretability we removed the negative flanker effects from the analyses below, but return to the issue at the end of the results’ section because it concerns removal of a non-neglible amount of data (25%).

To test if the two working memory and the two attention tasks showed an effect of bilingualism, a multiple linear regression analysis was performed on each of the four different dependent variables. The distribution of the Sky Search deviated strongly from normality (skewness = 1.76, kurtosis = 3.74) and for this reason a log-transformation was applied which improved the distribution (skewness = 0.25, kurtosis = 1.78). We included age, non-verbal IQ scores, and parental education as covariates. In order to control for level of Dutch, which was the language of instruction, PPVT scores were included as a covariate as well. A binary variable Group (monolingual versus bilingual) was included to evaluate the effect of bilingualism. The predictors were entered simultaneously. **Table 5** shows correlations between the background measures (age, non-verbal intelligence, SES, and PPVT) and between the dependent variables (backward Digit span, backward Dot matrix, Sky Search, positive flanker effect). The correlations show that the two working memory tasks correlate with each other and with the two attention tasks, whereas the two attention tasks do not correlate with each other. The outcomes of the regression analyses are summarized in **Table 6**.

**Table 5:** Correlations between the background variables age, NVIQ, SES, and receptive vocabulary and the dependent variables backward Digit Span task, backward Dot Matrix task, Sky Search task, and the positive flanker effect.

	Age	NVIQ	SES	PPVT	Backward Digit Span	Backward Dot Matrix	Sky Search	Positive flanker effect
Age		-0.10	-0.06	-0.18^∗^	0.30^∗∗^	0.32^∗∗^	-0.32^∗∗^	-0.24^∗∗^
NVIQ			0.12	0.24^∗∗^	0.25^∗∗^	0.28^∗∗^	-0.11	-0.16
SES				0.15^∗^	0.05	0.08	0.08	-0.08
PPVT					0.16^∗^	0.17^∗^	-0.10	-0.22^∗^
Backward DS						0.37^∗∗^	-0.22^∗∗^	-0.26^∗∗^
Backward DM							-0.29^∗∗^	-0.32^∗∗^
Sky Search								0.12

*NVIQ, standardized non-verbal intelligence score; SES, socioeconomic status; PPVT, Peabody Picture Vocabulary Test (Dutch receptive vocabulary, standardized score); ^∗^*p* < 0.05, ^∗∗^*p* < 0.01. Note that the correlations for the positive flanker effect are based on a somewhat smaller sample size, because the children with negative flanker effects are excluded.*

**Table 6:** Multiple regression models for the backward Digit Span task, backward Dot Matrix task, Sky Search task, and the positive flanker effect.

	Backward Digit Span		Backward Dot Matrix		Sky Search		Positive flanker effect	
	β	*p*	β	*p*	β	*p*	β	*p*
Age	0.35	<0.001	0.38	<0.001	-0.35	<0.001	-0.27	0.001
NVIQ	0.25	0.001	0.27	<0.001	-0.10	0.16	-0.14	0.11
SES	0.03	0.72	0.04	0.61	0.12	0.11	-0.03	0.70
PPVT	0.15	0.05	0.18	0.02	-0.20	0.01	-0.23	0.01
Group	-0.06	0.45	0.03	0.66	-0.19	0.01	-0.05	0.55

	Adjusted *R*^2^ = 0.17^∗∗∗^		Adjusted *R*^2^ = 0.21^∗∗∗^		Adjusted *R*^2^ = 0.15^∗∗∗^		Adjusted *R*^2^ = 0.11^∗∗∗^	

*NVIQ, standardized non-verbal intelligence score; SES, socioeconomic status; PPVT, Peabody Picture Vocabulary Test (Dutch receptive vocabulary, standardized score); ^∗∗∗^*p* < 0.001.*

Age had an effect on all dependent variables in the expected direction: a higher age predicted better performance. Non-verbal intelligence predicted both working memory outcomes, but it did not predict attention. Parental education had no effect on any of the variables, but this could be due to a relatively limited range and lack of variation, as indicated by the means and standard deviations in **Table 1**. Receptive vocabulary had an effect on all measures in interpretable directions: children with larger receptive vocabulary scores in Dutch performed better on the working memory and attention tasks. Group affected performance on the Sky Search, with a better score for bilinguals compared to monolinguals. Normal probability plots of the residuals indicated that the residuals are normally distributed for the backward Dot Matrix and Sky Search tasks. For the backward Digit Span task and flanker effect, the residuals showed a slight right-skew.

To determine which bilingual groups outperformed the monolinguals on the Sky Search, an ANCOVA with age, non-verbal IQ scores, SES, and PPVT as covariates, the four-level variable Group as the independent variable and the Sky Search as outcome variable was performed. This analysis did not reach statistical significance [*F*(3,167) = 2.50, *p* = 0.06, $\eta_{p}^{2}$ = 0.04].

### Exploring the Role of Proficiency in the Non-dutch Language

The data in **Table 4** indicate that all three bilingual groups score, on average, better than the monolinguals on the Sky Search, but the Polish bilinguals show the least indications they may benefit from their bilingualism. The context in which the Polish children develop the non-Dutch language is generally less favorable than the bilingual immersion context of the Frisian and Limburgish children. To explore if a lack of Polish proficiency affected the outcomes, we divided the Polish sample into two equally sized subgroups with, according to parental report, low Polish skills (0–0.67; LPS) and high Polish skills (0.72–1.0; HPS). We then validated the binary split by comparing the Polish receptive vocabulary scores in the two groups. The LPS group scored considerably lower (*M* = 57, *SD* = 13.5; raw scores) on Polish receptive vocabulary than the HPS group (*M* = 70, *SD* = 12; raw scores) [*F*(1,42) = 11.60, *p* = 0.001, $\eta_{p}^{2}$ = 0.22]. The LPS group received less Polish input before age 4 compared to the HPS group [*F*(1,42) = 4.50, *p* = 0.04, $\eta_{p}^{2}$ = 0.10], and current use of Polish was lower in the LPS group than in the HPS group [*F*(1,42) = 6.59, *p* = 0.01, $\eta_{p}^{2}$ = 0.14]. Dutch receptive vocabulary was the same in both groups [*F*(1,42) = 0.48, *p* = 0.49, $\eta_{p}^{2}$ = 0.01].

We reran the ANCOVA for the Sky Search, with age, non-verbal IQ scores, SES, and PPVT as covariates and the four-level variable Group as the independent variable. The Polish-Dutch group was limited to the HPS subgroup, which scored on average 5.14 (*SD* = 1.62) on the Sky Search. This time the effect of Group was significant [*F*(3,146) = 2.92, *p* = 0.036, $\eta_{p}^{2}$ = 0.06]. Bonferroni pairwise comparisons indicated that the Frisian-Dutch children (*p* = 0.01), Limburgish-Dutch children (*p* = 0.03) and the Polish-Dutch children (*p* = 0.03) outperformed the monolinguals. Visual inspection of the distribution revealed that one child in the Limburgish sample showed an out-of-range value. In order to test if this child did have a disproportional effect on the outcomes, we ran the analysis using bootstrapping. Based on 1,000 bootstrap samples, we found a significant difference between the Frisian-Dutch and the monolingual group, 95% CI [0.05–0.34], and also between the Polish-Dutch group and the monolinguals, 95% CI [0.03–0.42]. The difference between the Limburgish-Dutch group and the monolingual group did not reach significance, 95% CI [-0.02 to 0.35]. Running the ANCOVA for the Sky Search with age, non-verbal IQ scores, SES, and PPVT as covariates and the four-level variable Group as the independent variable, including only the Polish LPS subgroup, we also observed a significant effect of Group [*F*(3,146) = 2.93, *p* = 0.036, $\eta_{p}^{2}$ = 0.06]. However, Bonferroni pairwise comparisons indicated that the Frisian-Dutch children (*p* = 0.01) and the Limburgish-Dutch children (*p* = 0.04) outperformed the monolinguals, but the Polish-Dutch children did not (*p* = 0.85). Pairwise comparisons using bootstrapping to reduce the bias caused by the extreme value in the Limburgish sample revealed that the Frisian-Dutch children performed better than the monolinguals, 95% CI [0.05–0.34], but the Limburgish-Dutch, 95% CI [-0.01 to 0.34], and the Polish-Dutch groups, 95% CI [-0.19 to 0.22], did not differ significantly from the monolinguals. Note furthermore, that including only the Polish HPS group did not affect the outcomes of the two working memory tasks and the flanker effect, even though the HPS group also scored relatively well on these tasks. Moreover, including only the 50% children who were, according to parental report, the most skilled in Frisian and Limburgish did not result in a larger general effect of bilingualism nor did it lead to a significant effect in the Limburgish sample.

### Negative Flanker Effects

As indicated above, 25% of the flanker data showed a negative flanker effect, instead of the expected positive flanker effect. Because this is a non-negligible amount of data, it is important to investigate if these data are distributed randomly. The children with negative and positive flanker effects turned out to be very similar in many respects. They did not differ in age [*F*(1,174) = 0.22, *p* = 0.64, $\eta_{p}^{2}$ = 0.001], non-verbal intelligence [*F*(1,174) = 0.11, *p* = 0.74, $\eta_{p}^{2}$ = 0.001], SES (*U* = 2988, *p* = 0.77), and gender [χ^2^(1) = 0.03, *p* = 0.86]. Interestingly, relatively more monolinguals than bilinguals showed a negative flanker effect [χ^2^(1) = 7.92, *p* = 0.008]. **Table 7** shows the distribution of the negative and positive flanker effects across the different groups.

**Table 7:** Negative and positive flanker effect (in ms) distribution in groups.

	Negative flanker effect (*n* = 44)		Positive flanker effect (*n* = 132)	
	*n*		*n*	
Monolingual	18	-79.37 (70.77)	26	210.38 (161.64)
Bilingual	26	-120.18 (155.28)	106	199.31 (166.41)
Frisian	8	-38.19 (64.30)	36	183.03 (180.91)
Limburgish	11	-182.02 (207.95)	33	175.38 (143.33)
Polish	7	-116.70 (99.52)	37	236.49 (168.75)

In the monolingual sample, 41% of the children showed a negative flanker effect (ranging between -13.53 and -257.63). In the three bilingual samples, this percentage was lower: 18% of the Frisian children (range between -1.95 and -116.63), 25% of the Limburgish children (range between -2.25 and -636.66) and 16% of the Polish children (range between -10.49 and -299.58). The difference with monolinguals was significant for the Frisian [χ^2^(1) = 5.46, *p* = 0.03] and the Polish children [χ^2^(1) = 6.76, *p* = 0.02], but did not reach significance in the Limburgish group [χ^2^(1) = 2.52, *p* = 0.17].

## Discussion and Conclusion

In this study we compared three bilingual groups of 6–7-year-old children with a monolingual control group on two working memory (verbal, visuospatial) and two attention measures (selective attention, interference suppression). The three bilingual groups differed in sociolinguistic setting: two of the bilingual groups were exposed to a regional language (Frisian, Limburgish) in addition to the nation’s dominant language (Dutch), and the third bilingual group consisted of children exposed to a migrant language (Polish) besides Dutch. The inclusion of different bilingual groups is relevant in light of the growing awareness that contextual factors moderate the effect of bilingualism on cognitive development (Green and Abutalebi, 2013; Scaltritti et al., 2015; for an overview see the research topic of Yoshida et al., 2015, and the Bilingualism Forum, 2015 in Cortex). To exclude confounding effects, the four groups were matched on age, non-verbal intelligence, SES, and gender. The regional bilingual language groups and the monolingual group were culturally comparable. Multiple regression analyses, in which all bilinguals were grouped together and compared with the monolinguals, demonstrated that bilinguals outperformed monolinguals on selective attention. Pairwise comparisons of the separate bilingual groups and the monolingual controls suggest that the overall effect of bilingualism on selective attention was carried by the Frisian-Dutch children and the more bilingually proficient Polish-Dutch children. On the Flanker task, which tests the ability to suppress interference, monolingual and bilingual groups differed in the extent to which the incongruent flanking fish led to a slower or a faster response. The working memory tasks showed no effects of bilingualism.

These outcomes support the hypothesis that bilingualism influences the development of attention and confirm that effects of bilingualism on cognition are found across different sociolinguistic settings, that is, children acquiring a regional language (Costa et al., 2008, 2009; Hernández et al., 2013; Lauchlan et al., 2013; Garraffa et al., 2015; Antoniou et al., 2016; Bosma et al., 2017) and children learning a migrant language (Carlson and Meltzoff, 2008; Engel de Abreu et al., 2012; Blom et al., 2014). The data also indicate that the positive effect of bilingualism on the Sky Search is small, elusive, and dependent on sampling and task. For instance, the difference between monolinguals and bilinguals was rather robust in the Frisian sample. In contrast, in the Limburgish sample, the effect did not survive an analysis in which bootstrapping was used. Also, the Polish-Dutch group showed a positive effect of bilingualism, but only if 50% of the children with highest proficiency in Polish were included, confirming that a certain level of bilingual proficiency is required for the cognitive benefits to develop (Carlson and Meltzoff, 2008; Bialystok and Barac, 2012; Poarch and van Hell, 2012; Videsott et al., 2012; Blom et al., 2014; Antoniou et al., 2016; Crivello et al., 2016; Weber et al., 2016). Lastly, the enhancing effect of bilingualism emerged in the Sky Search, which measured selective attention, but not in the Flanker task, which measured attentional control and specifically the ability to suppress interference, and the two working memory tests.

We were not surprised to find that the expected effect of bilingualism emerged in only one task. Much previous research focused on interference suppression, guided by the hypothesis that bilingualism affects inhibitory control because bilinguals continuously need to suppress the interfering language (Green, 1998; Bialystok et al., 2004). However, Paap and Greenberg (2013) as well as Ross and Melinger (2016) showed that the findings on classic inhibition tasks, including Flanker tasks similar to the one we used in our study, are mixed. An increasing number of studies has observed bilingual advantages in tasks testing working memory (Vejnović et al., 2010; Morales et al., 2013; Blom et al., 2014; Kaushanskaya et al., 2014; Delcenserie and Genesee, 2016). However, also with respect to working memory, the outcomes of research are mixed (see Discussion; Calvo et al., 2016). Chung-Fat-Yim et al. (2017, p. 370) suggest that the mixed results on inhibition and working memory tasks in previous research might be “because those components do not define crucial differences between monolingual and bilingual cognition.” Instead, they hypothesize that selective attention is primarily influenced by bilingualism, a claim that finds support in our study in a surprisingly consistent way: three bilingual groups scored better than monolinguals on the same selective attention test. In two of the groups (Frisian, proficient Polish) this difference reached statistical significance.

Besides the Sky Search task, a Flanker task was used. In a previous study bilingual Portuguese-Luxembourgish children outperformed Portuguese monolingual children on this task (Engel de Abreu et al., 2012). In our study this finding was not replicated, but a comparison of the positive and negative flanker effects revealed a different effect of bilingualism. Relatively many monolinguals showed a negative flanker effect, indicating that they were faster in the incongruent than in the congruent condition. The difference between monolinguals and bilinguals in the relative frequency of negative flanker effects was significant for the Frisian and Polish children, but, again, did not reach statistical significance in the Limburgish sample. To our knowledge, negative flanker effects have not been reported explicitly in the literature on bilingualism, despite the fact that the “direction of the flanker effect has been a topic of some controversy” (Rouder and King, 2003, p. 288). It is conceivable that the negative flanker effects contribute to the elusiveness of the effects of bilingualism in studies using Flanker tasks (Paap and Greenberg, 2013; Ross and Melinger, 2016).

Rouder and King (2003) ascribe the negative flanker effects to contrast enhancement in lower order perceptual processes early in stimulus processing. Positive flanker effects, in contrast, may reveal response competition in the response selection processes, which takes place later in stimulus processing. Possibly, bilingual children filter out less and attend to more information in their environment, because they are used to attending to many cues for deciding which language to use in their everyday life. The simultaneous processing of contrasting stimuli may, moreover, be common for bilingual children. This happens, for instance, when they interact in one of their languages while a movie is playing in the other language or when they listen to one language while reading the other language as can happen in the case of subtitles. More experience with the simultaneous processing of contrasting information might reduce the effect of contrast enhancement.

The relatively few negative flanker effects in the bilingual sample indicate that the bilingual children in our study more often show response competition which, in turn, demonstrates that they attend to the incongruent flanking fish more than the monolingual children did. However, it was not the case that the bilingual children were more bothered by the incongruent flanking fish, as shown by the absence of a difference when positive flanker effects were compared across monolinguals and bilinguals. Moreover, in the Sky Search task, in which the children were asked to focus on the contrast and compare two adjacent space ships to decide on their similarity, the bilingual children outperformed the monolinguals. This shows that when the children’s task is to detect a contrast between stimuli, instead of ignoring interference from contrasting stimuli (as in the Flanker test), bilingual children excel. This supports the hypothesis that bilingual experiences change the way attention is directed to the environment and enhance selective attention (Bialystok, 2015; Chung-Fat-Yim et al., 2017).

In our study two groups of regional language users were tested. In the Frisian sample, the effects of bilingualism were more robust than in the Limburgish sample, both for the Sky Search and for the flanker effect. Parents’ ratings of their children’s language skills in the two languages indicate that the Limburgish parents rated their children’s skills in Limburgish rather low, which may suggest that a lack of bilingual proficiency is related to the less robust effect of bilingualism in the Limburgish sample. However, including only the 50% of the children with, according to parental report, high Limburgish proficiency did not alter the outcomes, suggesting that bilingual proficiency does not play a role. We suggested that teaching Frisian as a subject in school, which is obligatory for at least 1 h per week, may lead to more language separation in the Frisian context. A higher degree of language separation may be linked to cognitive effects of bilingualism (Green and Abutalebi, 2013). However, in many schools Frisian is also used as a language for instruction, like Dutch. Functional overlap between the two languages may have the opposite effect and lead to less separation instead of more, although the direction of the effect may be dependent on specific language use strategies that could vary from school to school and from teacher to teacher (e.g., use of specific days for each language, different classrooms, different subjects, or use of both languages for the same subject, in the same classroom, and at the same day).

Interestingly, recent research comparing *tweets* in Fryslân and Limburg suggests that Limburgish is more often used in tweets than Frisian, but also that Limburgish is more frequently mixed with Dutch (Trieschnigg et al., 2015), which is consistent with the findings by Giesbers (1989) showing frequent mixing between Limburgish and Dutch. Frequent mixing in the Limburgish context is, moreover, supported by the study of Francot et al. (in press) who observed that in a Limburgish word naming task, children used many mixed forms that had characteristics of both Limburgish and Dutch. If these cross-regional differences in language use are representative of the children in our sample, the Limburgish parents may have rated their children’s Limburgish relatively low because of frequent mixing with Dutch or because their children’s language use is not in accordance with the parents’ normative idea of how a dialect should be spoken. Moreover, frequent mixing may suggest that between-language links are stronger for Limburgish and Dutch than for Frisian and Dutch, explaining why the effect of bilingualism on attention is more robust for the Frisian than for the Limburgish children (Gathercole et al., 2014). If in the Limburgish context frequent mixing is indeed more common than in the Frisian context, the pattern is also in line with Green and Abutalebi (2013) who predict that dense code-mixing behavior is less associated with cognitive control than bilingual behavior in which different languages are used in different environments or both languages are used but with different speakers. The parental questionnaire in our study provided information on language use in the home environment and no information was available on patterns of language use outside of this context. For this reason, we refrained from investigating the influence of language separation and overlap. We do, however, consider this an important venue for future research on the cognitive effects of bilingualism.

This study revealed that cognitive effects of bilingualism are found in children who become bilingual because they are exposed to a regional language, in addition to the national language, and in children who become bilingual because (one of) their parents migrated. Comparisons of different tasks show that bilingual experiences primarily influence how children direct their attention to the environment: it appears that they consider more information to be potentially task-relevant and they are relatively successful at using this information in a task in which they need to focus and attend selectively, i.e., compare two paired stimuli and decide on their similarity. The findings in this study demonstrate that for migrant children, proficiency in the home or migrant language is essential for cognitive advantages to develop and suggest that the cognitive effects for regional language speakers are modulated by differences in sociolinguistic settings.

## Author Contributions

All authors were involved in the conception and design of the study. EBl wrote the introduction, results and discussion and carried out the statistical analyses. EE contributed to the literature review and finalizing of the manuscript. TB and EBo drafted the method section and contributed significantly to the collection, processing and analysis of the monolingual and Frisian data respectively. LC enabled the collection of the Limburgish data. TB, EBo, LC, and EE revised the draft for critical content.

## Conflict of Interest Statement

The authors declare that the research was conducted in the absence of any commercial or financial relationships that could be construed as a potential conflict of interest.
